# Supplementary figures and images for: Canonical and Cross-reactive Binding of NK Cell Inhibitory Receptors to HLA-C Allotypes Is Dictated by Peptides Bound to HLA-C
Source: Front Immunol. 2017 Mar 14;8:193. doi: 10.3389/fimmu.2017.00193 (PMC5348643; doi:10.3389/fimmu.2017.00193)

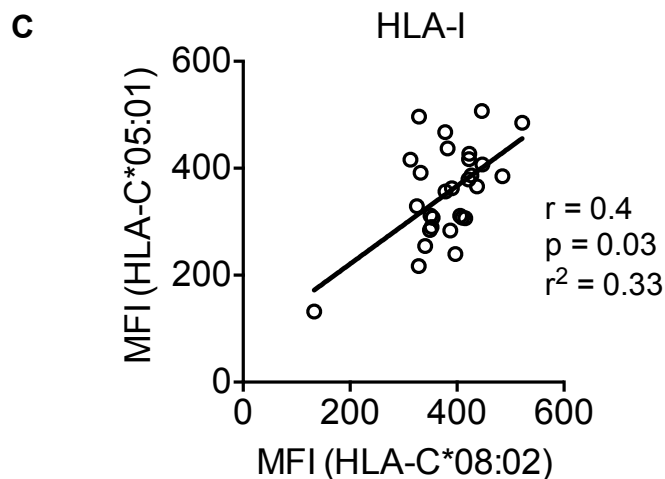

Supplement: Additional File S1 — Sequence alignment of HLA-C*05:01 and HLA-C*08:02, generation of TAP-deficient cell line expressing HLA-C*05:01 and correlation of HLA-I stabilization. (A) Amino acid alignment of HLA-C*05:01 and HLA-C*08:02. Box indicates positions 77–80. (B) Expression of ICP47 (via IRES-GFP) and HLA-C in 221–C*05:01 cells transfected with pzLRS–ICP47-IRES-GFP. After drug selection, 221–C*05:01–ICP47 cell line was cloned by limiting dilution. (C) Correlation of HLA-I stabilization. HLA-C*05:01 against HLA-C*08:02 (Spearman). [file Image_1.pdf]

**Additional file 3. HLA-C\*05:01 is a functional ligand for KIR2DL1, KIR2DL2 and KIR2DL3.**

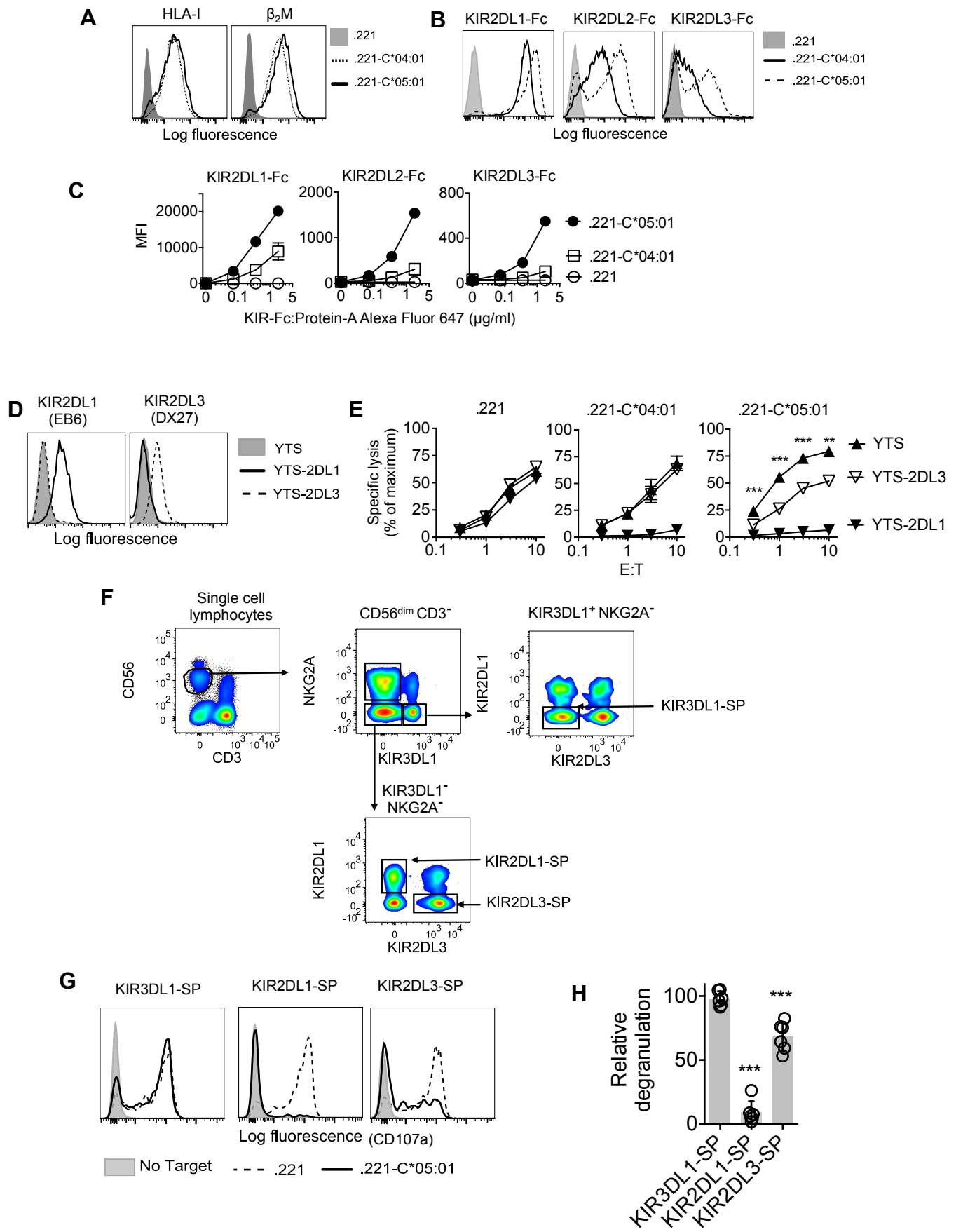

Supplement: Additional File S3 — HLA-C*05:01 is a functional ligand for KIR2DL1, KIR2DL2 and KIR2DL3. (A) Expression of HLA-I and β2 microglobulin on 221, 221–C*04:01 and 221–C*05:01 cells. (B,C) KIR2DL1-Fc, KIR2DL2-Fc, and KIR2DL3-Fc binding to 221, 221–C*04:01, and 221–C*05:01 cells as determined by flow cytometry. (C) Mean MFI and SEM are shown from three independent experiments. (D) Expression of KIR2DL1 and KIR2DL3 on YTS, YTS-2DL1, and YTS-2DL3 NK cells. (E) Specific lysis of 221, 221–C*04:01, and 221–C*05:01 cells by YTS, YTS-2DL1, and YTS-2DL3 NK cells at different effector–target ratios (E:T). Mean and SEM of three independent experiments are shown. **p < 0.01 and ***p < 0.001 by Student’s t-test. (F) Flow cytometry gating strategy to identify KIR SP (KIR-SP) NK cells. CD56dim CD3− lymphocytes were identified from PBMCs. KIR2DL3-SP NK cells were identified as KIR3DL1− and NKG2A−, KIR2DL1− NK cells from A/A KIR haplotype donors. (G) Flow cytometry histograms showing degranulation (CD107a at the cell surface) of KIR3DL1 SP, KIR2DL1-SP, and KIR2DL3-SP NK cells in response to 221, 221–C*05:01, or no target cells. (H) Degranulation of KIR-SP NK cells in response to 221 and 221–C*05:01 cells for N = 5 A/A KIR haplotype donors. Degranulation is shown relative to 221. ***p < 0.001 by Student’s t-test. [file Image_3.pdf]
