# Supplementary material for: Canonical and Cross-reactive Binding of NK Cell Inhibitory Receptors to HLA-C Allotypes Is Dictated by Peptides Bound to HLA-C
Source: Front Immunol. 2017 Mar 14;8:193. doi: 10.3389/fimmu.2017.00193 (PMC5348643; doi:10.3389/fimmu.2017.00193)
Supplement: Additional File S2 — HLA-C*05:01 eluted peptide sequences and relative abundance. The relative abundance: + = 0–29 copies/cell, ++ = 30–59 copies/cell, +++ = 60–100 copies/cell, and ++++ = >100 copies/cell. [file Image_2.pdf]

Additional file 2. HLA-C*05:01 eluted peptide sequences

Peptide	Sequence	Relative abundance	Protein Source	Uniprot Number
P2	IIDKSGSTV	++	AP-3 complex subunit mu-1	Q9Y2T2
P3	VGDKPVSNF	+	protein unc-119 homolog B	Q13432
P4	AVDGSGTKF	+	ribosome biogenesis protein WDR12	Q9GZL7
P6	IVDRGSTNL	+	alpha-adducin	P35611
P7	YVDEHGTRL	++	proteasome subunit beta type-8	P28062
P8	GSDPRVTQL	++++	RILP-like protein 2	Q969X0
P9	IVDRPVTLV	+++	NADH dehydrogenase [ubiquinone] 1 beta subcomplex subunit 10	Q9P0J0
P10	IVDKSGRTL	++	methionine synthase	Q99707
P11	AGDDAPRAV	+++	actin OR POTE ankyrin domain family member	P60709/ Q6S8J3
P12	VSDQANHVL	++	kinesin-like protein KIF21A	Q7Z4S6
P13	ASDHAPHTL	+	CAD protein	P27708
P14	VGDPHTVTV	+	bromodomain-containing protein 8	O60885
P15	KSDERPVHI	+	E3 ubiquitin-protein ligase TRIM33	Q9UPN9
P16	ASDDGTVRI	++	WD repeat-containing protein 26	Q969H0
P17	SIDKTGENF	+	40S ribosomal protein S4, X isoform	P62701
P18	TSDDVAKEF	+++	protein unc-13 homolog C	O14795
P19	SADSKPIDV	+	aminoacyl tRNA synthase complex-interacting multifunctional protein 1	Q12904
P20	TAEHPGTRL	++	DNA topoisomerase 2-binding protein 1	Q92547
P21	MADRGEARL	++	recQ-mediated genome instability protein 2	Q96E14
P22	AADFEIGHF	+	nucleosome assembly protein 1-like 1-A	Q4U0Y4
P23	NADGKIISL	+++	bifunctional glutamate/proline--tRNA ligase	P07814
P24	AADGKGVVV	++	60S ribosomal protein L28	P46779
P25	AADKIHSSF	++++	plasminogen activator inhibitor 2	P05121
P26	NADAIVVKL	+++	staphylococcal nuclease domain-containing protein 1	Q7KZF4
P27	NADTVSSKL	+++	AP-2 complex subunit beta	P63010
P28	SAEKAPVSV	+++	transforming acidic coiled-coil-containing protein 1	O75410
P29	QGDAVVLKI	+++	immunoglobulin superfamily member 8 precursor	Q969P0
P30	AADHYSQQM	+	guanylate-binding protein 4	Q96PP9
**Length**	**Sequence**	**Relative abundance**	**Protein Source**	**Uniprot Number**
8	SSDGTVKI	+	WD40 repeat-containing protein SMU1	Q2TAY7
8	NADEARSL	+++	eukaryotic translation initiation factor 1A, X/Y-chromosomal	P47813/ O14602
8	SADEVQRF	++++	heterogeneous nuclear ribonucleoprotein H	P31943
8	VADSKTLL	+++	ribonuclease H2 subunit A	O75792
8	KVDQEVKL	+++	proteasome activator complex subunit 3	P61289
8	AADGSVKL	++	U4/U6 small nuclear ribonucleoprotein Prp4	O43172
8	ASDGTVRL	++++	heterogeneous nuclear ribonucleoprotein H3	P31942

Length	Sequence	Relative Abundance	Protein Source	Uniprot Number
8	SADGTVRI	++++	striatin (3 or 4)	Q13033/ Q9NRL3
8	SADKTVAL	+++	histone-binding protein RBBP7	Q16576
8	AQDQPVKM	+	glycylpeptide N-tetradecanoyltransferase 1	P30419
8	HTDTTVKF	+++	DNA topoisomerase 2-alpha	P11388
8	HVDAHATL	++	ras GTPase-activating protein-binding protein 1	Q13283
8	VADKFTEL	++	serine/threonine-protein phosphatase 2A 65 kDa regulatory subunit A alpha isoform	P30153
8	NSDRPGVL	++	coronin-7	P57737
8	VADKHELL	++++	eukaryotic translation initiation factor 3 subunit H	O15372
8	TADKAVDL	++	DNA polymerase alpha catalytic subunit	P09884
8	ASDVHGNF	+	sorting nexin-17	Q15036
8	VADKDTVL	+	protein disulfide-isomerase A4	P13667
8	VADHIQKV	+	coatomer subunit gamma-1	Q9Y678
8	SADKYVKI	+	periodic tryptophan protein 1 homolog	Q13610
8	VIDEPVRL	++	Proliferation marker protein KI-67	P46013
8	VSDKDASL	++	C-myc promoter-binding protein	Q7Z401
9	SADDKTVRL	++++	serine-threonine kinase receptor-associated protein	Q9T3F4
9	SADKPLSNM	++++	poly [ADP-ribose] polymerase 1	P09874
9	VSDSGAHVL	++	protein asunder homolog	Q8NVM9
9	AADDSSVKL	+++	pre-mRNA-processing factor 19	Q9UMS4
9	AAEKKVVKI	++	SAP domain-containing ribonucleoprotein	P82979
9	KADNVGSAL	+	pyridine nucleotide-disulfide oxidoreductase domain-containing protein 1	Q8WU10
9	NAEQHVTQL	++	inner centromere protein	Q9NQS7
9	VAEHHVATL	++	cell division cycle protein 20 homolog	Q12834
9	SADELVTRI	+++	UBX domain-containing protein 4	Q92575
9	VADEQNRAL	+	E3 ubiquitin-protein ligase TRIM56	Q9BRZ2
9	VADQIVTKL	++	DNA topoisomerase 2-alpha	P11388
9	NADPQAVTM	++++	melanoma-associated antigen D2	Q9UNF1
9	VVDQGSTNL	+	gamma-adducin	Q9UEY8
9	RADsPVHM	++	Mediator of RNA polymerase II transcription subunit 26	O95402
9	VVDKPPSTM	++	gamma-adducin	Q9UEY8
9	SIDKAVTKL	+	polycomb protein SUZ12	Q15022
9	IVDVRPTLV	+++	1-phosphatidylinositol 3-phosphate 5-kinase	Q9Y2I7
9	KLDETGNSL	++	DNA topoisomerase 2-alpha	P11388
9	IIDKSGSTI	++	AP-3 complex subunit mu-2	P53677
10	KADGIVSKNF	+++	40S ribosomal protein S21	P63220
10	KADTVSKTEL	++++	septin-10	Q9P0V9
10	NAESGRGQVM	++++	interleukin-32	P24001
