# Supplementary material for: Canonical and Cross-reactive Binding of NK Cell Inhibitory Receptors to HLA-C Allotypes Is Dictated by Peptides Bound to HLA-C
Source: Front Immunol. 2017 Mar 14;8:193. doi: 10.3389/fimmu.2017.00193 (PMC5348643; doi:10.3389/fimmu.2017.00193)

Additional file 4. KIR2DL2 and KIR2DL3 binding to HLA-C*08:02 is more peptide selective than KIR2DL1 binding to HLA-C*05:01. Data normalized to HLA-I (KIF-Fc MFI/HLA-I MFI) for each peptide.

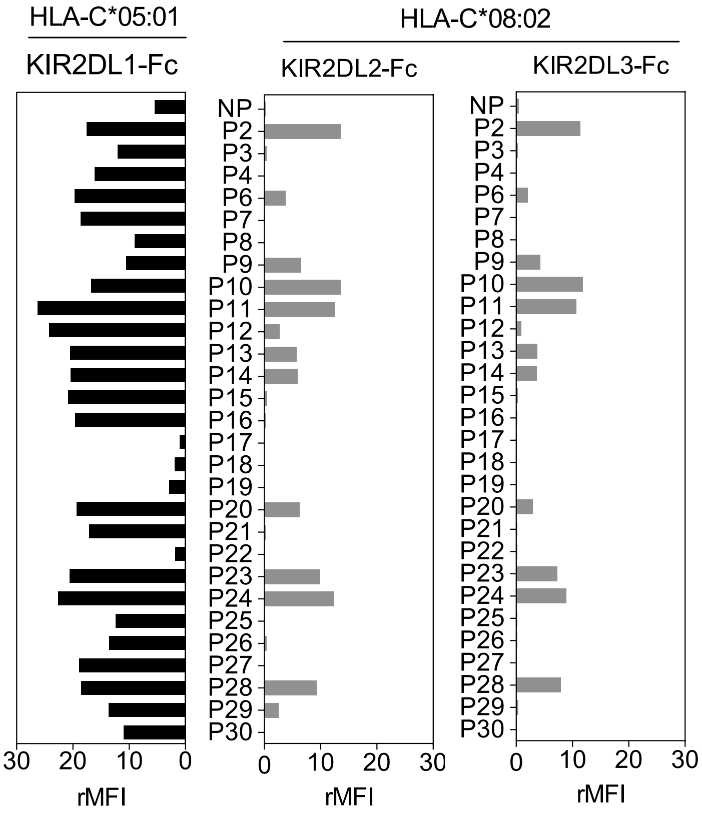

Supplement: Additional File S4 — KIR2DL2 and KIR2DL3 binding to HLA-C*08:02 is more peptide selective than KIR2DL1 binding to HLA-C*05:01. Data from Figure 2B normalized to HLA-I (KIF-Fc MFI/HLA-I MFI) for each peptide. [file Image_4.pdf]
