# Supplementary material for: Canonical and Cross-reactive Binding of NK Cell Inhibitory Receptors to HLA-C Allotypes Is Dictated by Peptides Bound to HLA-C
Source: Front Immunol. 2017 Mar 14;8:193. doi: 10.3389/fimmu.2017.00193 (PMC5348643; doi:10.3389/fimmu.2017.00193)

Additional file 5. HLA-C*05:01 HIV Gag peptides modulate canonical and cross-reactive binding of KIR2DL2 and KIR2DL3 to HLA-C*05:01(C2).

A

Peptide	Sequence
Gag_{50-58}	LLETPEGCL
Gag_{60-68}	IIEQIQPAI
$Gag_{305-313}$	RAEQATQDV
$Gag_{310-318}$	TQDVKNWMT
$Gag_{427-435}$	CTERQANFL

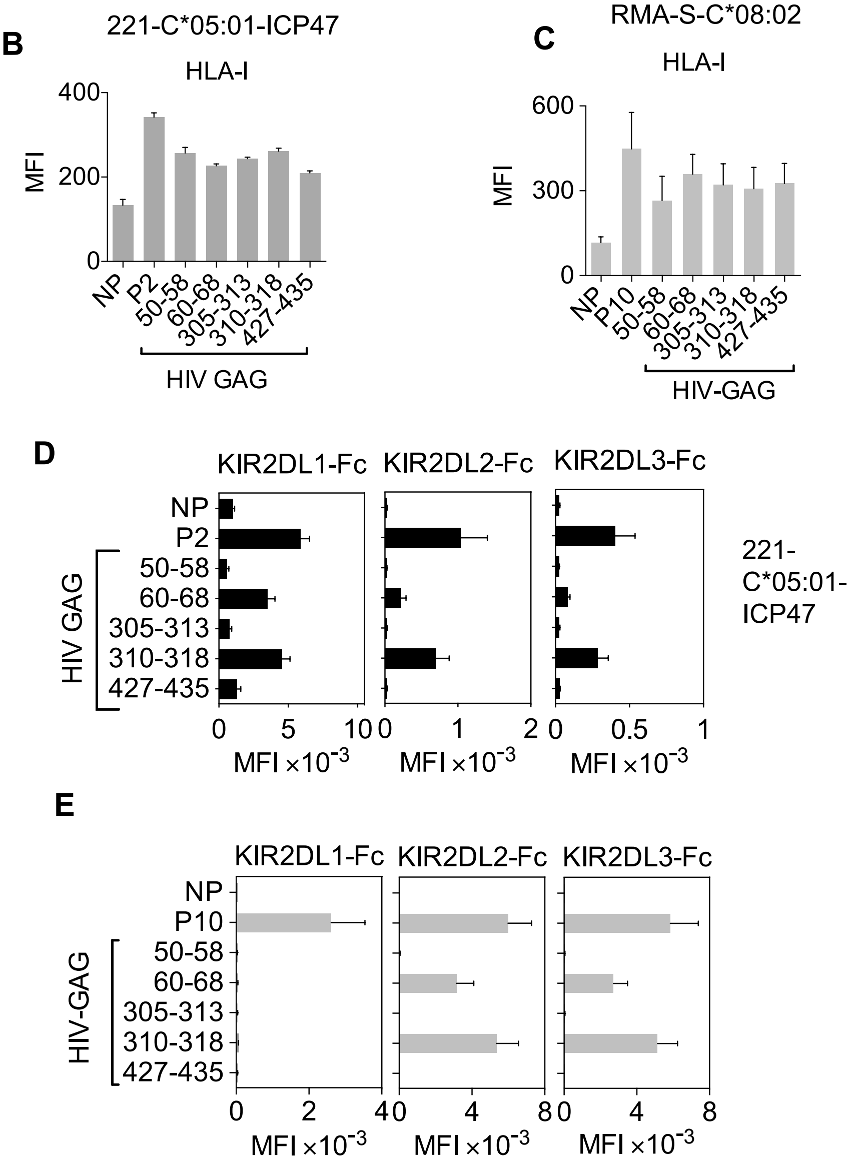

Supplement: Additional File S5 — HLA-C*05:01 HIV Gag peptides modulate canonical and cross-reactive binding of KIR2DL2 and KIR2DL3 to HLA-C*05:01(C2). (A) HLA-C*05:01 HIV Gag 9mer peptide sequences. (B) Expression of HLA-I on 221–C*05:01–ICP47 cells loaded with no peptide (NP), P2 and HIV Gag peptides 50–58, 60–68, 305–313, 310–318, and 427–435. Mean MFI and SEM of three independent experiments are shown. (C) Expression of HLA-I on RMA-S-C*08:02 cells loaded with NP, P2 and HIV Gag peptides 50–58, 60–68, 305–313, 310–318, and 427–435. Mean MFI and SEM of three independent experiments are shown. (D) KIR2DL1-Fc, KIR2DL2-Fc and KIR2DL3-Fc binding to 221–C*05:01–ICP47 cells loaded with NP, P2, or HIV Gag peptides, 50–58, 60–68, 305–313, 310–318, and 427–435. Mean MFI and SEM of three independent experiments are shown. (E) KIR2DL1-Fc, KIR2DL2-Fc, and KIR2DL3-Fc binding to RMA-S-C*08:02 cells loaded with NP, P2, or HIV Gag peptides, 50–58, 60–68, 305–313, 310–318, and 427–435. Mean MFI and SEM of three independent experiments are shown. [file Image_5.pdf]
