# Supplementary material for: Canonical and Cross-reactive Binding of NK Cell Inhibitory Receptors to HLA-C Allotypes Is Dictated by Peptides Bound to HLA-C
Source: Front Immunol. 2017 Mar 14;8:193. doi: 10.3389/fimmu.2017.00193 (PMC5348643; doi:10.3389/fimmu.2017.00193)

Additional file 6. Stabilization of HLA-I on TAP-deficient cells by peptides and cross-reactive binding of KIR2DL2 to peptide-loaded HLA-C*05:01 and KIR2DL1 to peptide-loaded HLA-C*08:02.

A

Peptide	Sequence
P2	IIDKSGSTV
P2-7A	IIDKSGATV
P2-8A	IIDKSGSAV
P2-AA	IIDKSGAAV
P2-GG	IIDKSGGGV
P2-8E	IIDKSGSEV
P2-EE	IIDKSGEEV
P2-AV	IIDKSGAVV
P2-AY	IIDKSGAYV
P2-FA	IIDKSGFAV
P2-LA	IIDKSGLAV
P2-KA	IIDKSGKAV
P2-RA	IIDKSGRAV
P10	IVDKSGRTL
P10-7E	IVDKSGREL
P10-AA	IVDKSGAAL
P11	AGDDAPRAV
P11-7A	AGDDAPAAV

B

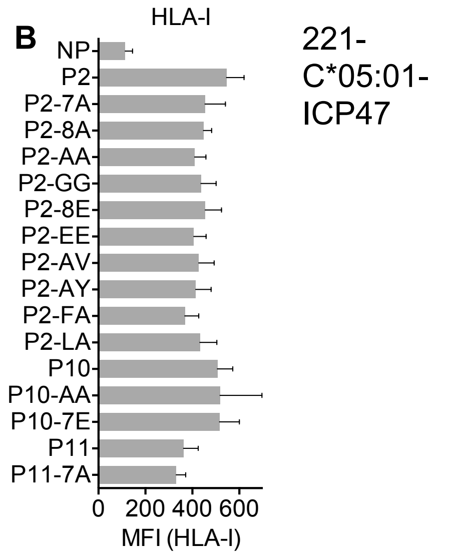

C

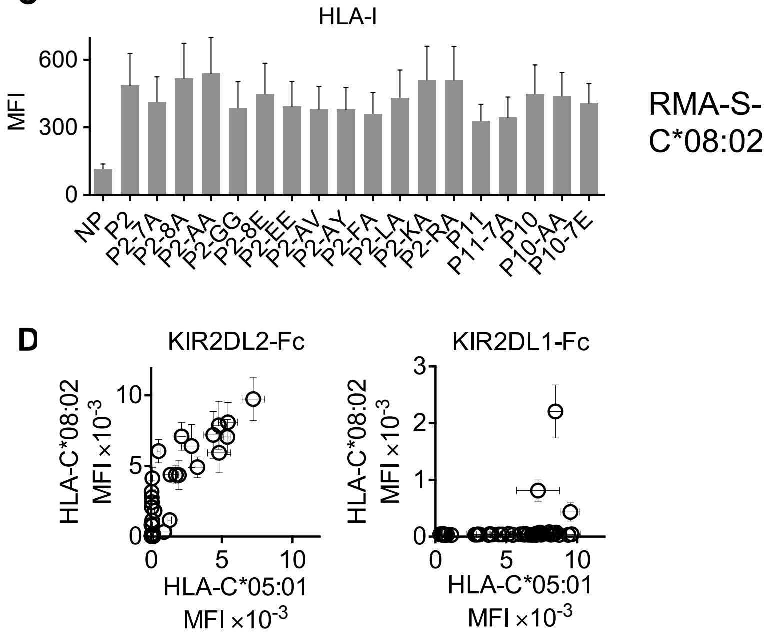

Supplement: Additional File S6 — Stabilization of HLA-I on TAP-deficient cells by peptides and cross-reactive binding of KIR2DL2 to peptide-loaded HLA-C*05:01 and KIR2DL1 to peptide-loaded HLA-C*08:02. (A) Sequences of peptides P2, P10, P11 and peptides with amino acid substitutions at positions 7 and 8. (B) HLA-I expression on 221–C*05:01–ICP47 cells after overnight culture in the presence of HLA-C*05:01 peptides P2, P10, P11, and amino acid-substituted peptides compared to cells with NP. Mean MFI and SEM of three independent experiments are shown. (C) HLA-I expression on RMA-S-C*08:02 cells after overnight culture in the presence of HLA-C*05:01 peptides P2, P10, P11, and amino acid-substituted peptides compared to cells with NP. Mean MFI and SEM of three independent experiments are shown. (D) KIR2DL2-Fc (left) and KIR2DL1-Fc (right) binding to HLA-C*05:01 is correlated with binding to HLA-C*08:02 in the presence of the same peptides. All peptides with amino acid substitutions are shown. [file Image_6.pdf]
