# Supplementary material for: Canonical and Cross-reactive Binding of NK Cell Inhibitory Receptors to HLA-C Allotypes Is Dictated by Peptides Bound to HLA-C
Source: Front Immunol. 2017 Mar 14;8:193. doi: 10.3389/fimmu.2017.00193 (PMC5348643; doi:10.3389/fimmu.2017.00193)

Additional file 7. Degranulation of KIR3DL1-SP NK cells in response to peptide-loaded TAP-deficient HLA-C*05:01 cells.

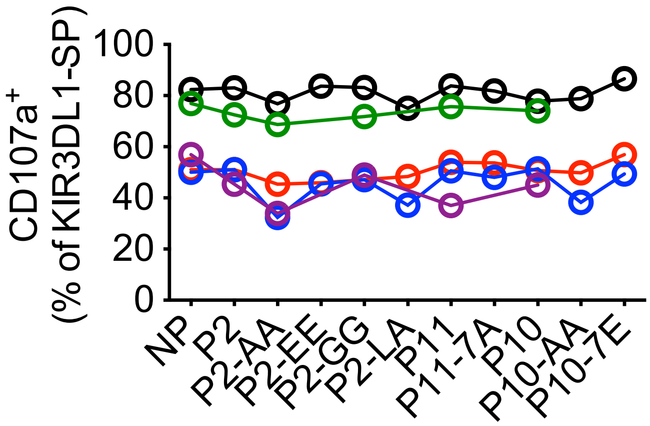

Supplement: Additional File S7 — Degranulation of KIR3DL1-SP NK cells in response to peptide-loaded TAP-deficient HLA-C*05:01 cells. KIR3DL1-SP NK cell CD107a expression (% positive) in response to 221–C*05:01–ICP47 cells loaded with NP, P2, P2-AA, P2-EE, P2-LA, P2-GG, P11, P11-7A, P10, P10-AA, and P10-7E. Individual donors are represented by different colors. [file Image_7.pdf]
