# Supplementary material for: Canonical and Cross-reactive Binding of NK Cell Inhibitory Receptors to HLA-C Allotypes Is Dictated by Peptides Bound to HLA-C
Source: Front Immunol. 2017 Mar 14;8:193. doi: 10.3389/fimmu.2017.00193 (PMC5348643; doi:10.3389/fimmu.2017.00193)

Additional file 8. KIR-Fc binding and functional responses of KIR+ NK cells to 221-C*08:02 and 221-C*08:02-ICP47 cells.

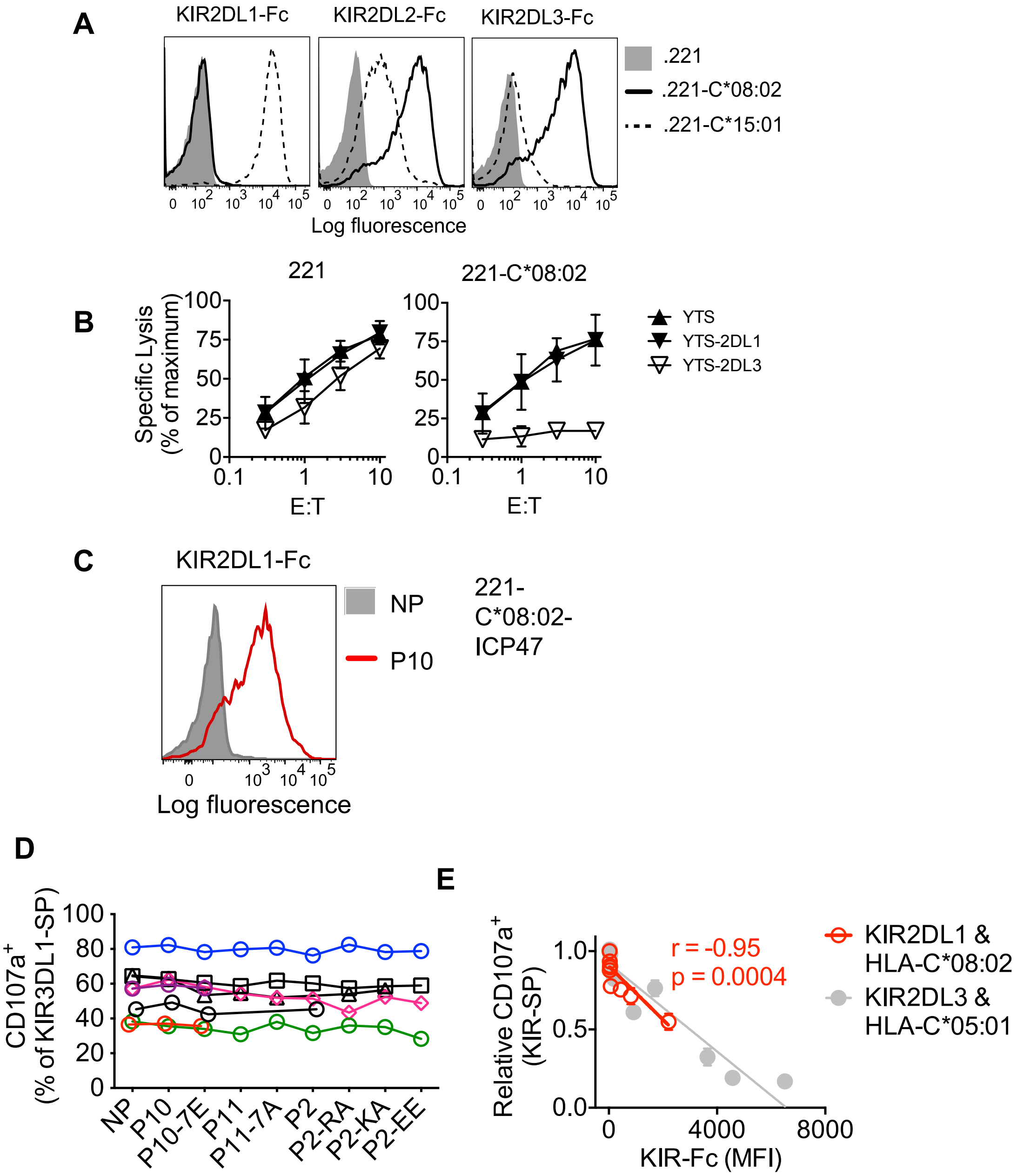

Supplement: Additional File S8 — KIR-Fc binding and functional responses of KIR+ NK cells to 221-C*08:02 and 221-C*08:02-ICP47 cells. (A) KIR2DL1-Fc, KIR2DL2-Fc, and KIR2DL3-Fc binding to 221, 221–C*08:02 and 221–C*15:01. (B) Specific lysis of 221 and 221–C*08:02 cells by YTS, YTS-2DL1, and YTS-2DL3 NK cells. (C) KIR2DL1-Fc binding to 221–C*08:02–ICP47 cells loaded with NP or P10. (D) KIR3DL1-SP NK cell CD107a expression (% positive) in response to 221–C*08:02–ICP47 cells loaded with NP, P2, P2-AA, P2-EE, P2-LA, P2-GG, P11, P11-7A, P10, P10-AA, and P10-7E. Individual donors are represented by different colors. (E) Overlay of cross-reactive KIR-SP NK cell responses and cross-reactive KIR-Fc binding to HLA-C*05:01 and HLA-C*08:02. [file Image_8.pdf]
